# Clinical Factors Associated with Body-weight Reduction Induced by Semaglutide 1.0 mg Weekly in Patients with Type 2 Diabetes Mellitus

**DOI:** 10.31662/jmaj.2024-0366

**Published:** 2025-03-28

**Authors:** Noboru Kurinami, Masafumi Takada, Kenji Ashida, Seigo Sugiyama, Akira Yoshida, Kunio Hieshima, Tomoko Suzuki, Fumio Miyamoto, Keizo Kajiwara, Katsunori Jinnouchi, Masatoshi Nomura, Hideaki Jinnouchi

**Affiliations:** 1 Diabetes Care Center, Jinnouchi Hospital, Kumamoto, Japan; 2 Division of Endocrinology and Metabolism, Department of Internal Medicine, Kurume University School of Medicine, Kurume, Japan; 3 Division of Cardiovascular Medicine, Diabetes Care Center, Jinnouchi Hospital, Kumamoto, Japan; 4 Division of Preventive Cardiology, Department of Cardiovascular Medicine, Kumamoto University Hospital, Kumamoto, Japan

**Keywords:** type 2 diabetes mellitus, obesity, glucagon-like peptide-1, weight reduction

## Abstract

**Introduction:**

We examined the clinical factors associated with a decrease in weight induced by weekly semaglutide in patients with type 2 diabetes mellitus (T2DM).

**Methods:**

Patients with T2DM who visited the Diabetes Care Center of Jinnouchi Hospital between June 2020 and October 2023 and were treated with semaglutide, 1.0 mg weekly, in addition to their ongoing medications were retrospectively registered. We measured body weight both before weekly administration of 1.0 mg semaglutide and 180 days after treatment and calculated the change in weight.

**Results:**

A total of 96 patients with T2DM were enrolled, with a mean body weight of 87.2 ± 17.1 kg and mean HbA1c of 7.3 ± 1.7% at baseline. The greater response group, defined as having 1.0 mg weekly semaglutide treatment-related weight reduction of more than 7.0%, comprised 23 patients (24.0%). Weekly 1.0 mg semaglutide treatment for 180 days significantly reduced body weight (−3.1 ± 4.8 kg, p < 0.001) and glycated hemoglobin (−0.39% ± 1.23%, p = 0.003). Multivariable logistic regression analysis found that pretreatment high-density lipoprotein (HDL)-cholesterol levels per 1.0 mg/dL (odds ratio [OR] 1.05; 95% confidence interval [CI] 1.01-1.09, p = 0.02) were independently and significantly associated with greater weight reduction after weekly 1.0 mg semaglutide treatment, while a switch from other glucagon-like peptide-1 receptor agonists (OR 0.31; 95% CI 0.11-0.87; p = 0.03) was independently and significantly associated with lesser weight reduction after weekly 1.0 mg semaglutide treatment. In receiver-operator characteristic analysis, the cutoff value of pretreatment HDL-cholesterol levels for the presence of greater response in weight reduction was 46 mg/dL (sensitivity 61%, specificity 62%; p = 0.03).

**Conclusions:**

Pretreatment HDL-cholesterol levels serve as important information for weekly treatment with 1.0 mg semaglutide in patients with T2DM and expectation of weight reduction.

## Introduction

Obesity is closely involved in the pathogenesis of type 2 diabetes mellitus (T2DM). The accumulation of visceral adipose tissue is a well-known risk factor for the development of insulin resistance, T2DM, and cardiovascular disease ^(1)^. The incidence of obesity is continually increasing and is recognized globally as an important clinical problem ^(2)^. In patients with T2DM, the treatment of diabetes accompanied by reduction of body fat and weight is potentially a valuable strategy that can lead to additional beneficial effects on the fundamental pathophysiology of T2DM.

Glucagon-like peptide-1 (GLP-1) reduces food intake via three pathways: the hypothalamus, the medullary area postrema, and afferent sensory neurons ^(3)^. Among GLP-1 receptor agonists (RAs), weekly semaglutide in particular has been reported to be effective in improving glucose metabolism and weight loss ^(4), (5)^. Weekly semaglutide treatment also induces early improvements in body composition in patients with T2DM ^(6)^. Thus, GLP-1 RAs are considered a suitable therapy for obese patients with T2DM. However, clinical factors associated with effective weight reduction remain unknown. To determine the efficacy of GLP-1 RA antidiabetic treatment in T2DM patients, we need to know the baseline clinical factors associated with GLP-1 RA in body-weight reduction.

In this study, based on results obtained from weekly treatment with 1.0 mg semaglutide for glucose reduction in T2DM, we examined the clinical factors associated with the decrease in weight induced by weekly semaglutide in patients with T2DM.

## Materials and Methods

### Subjects and protocol

**Figure 1.**
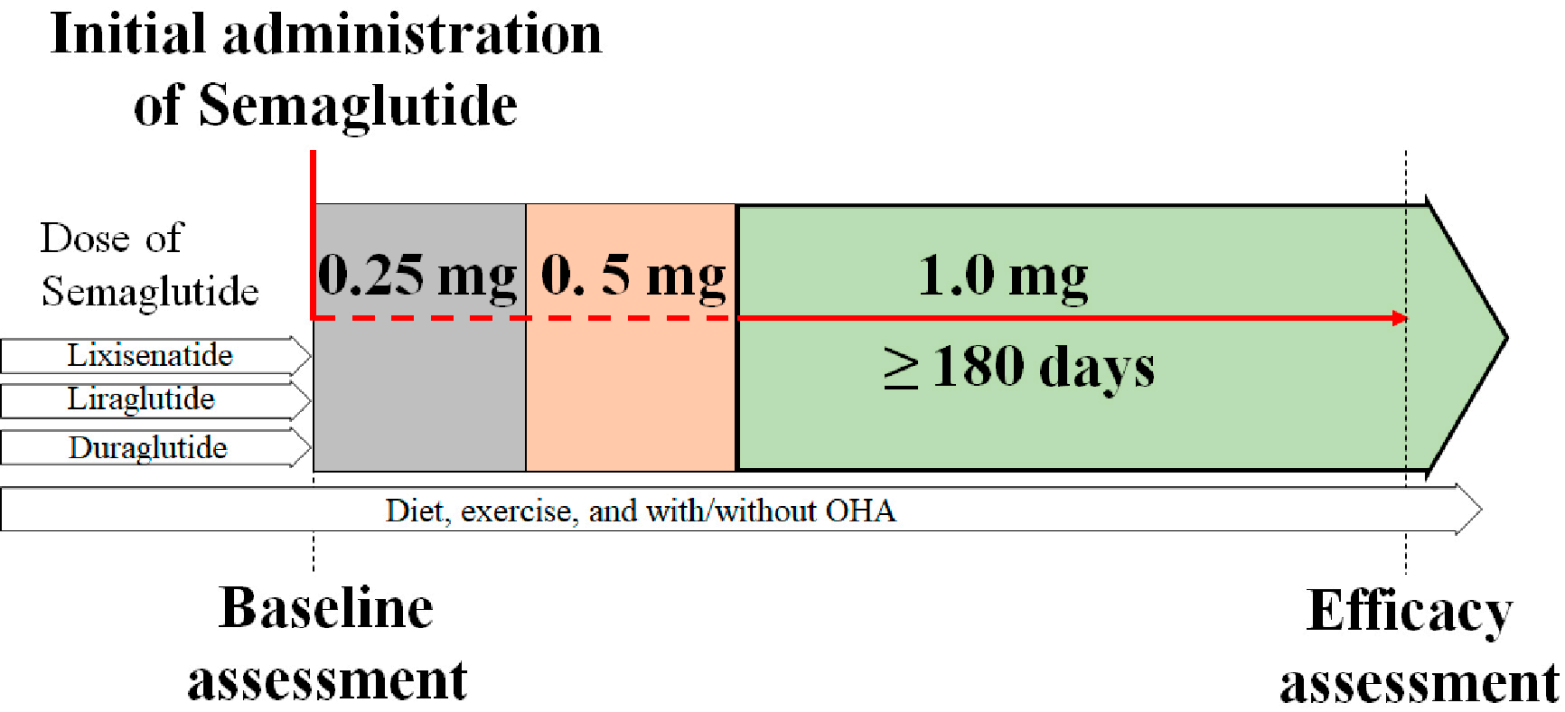
Flow Chart of This Study. Baseline assessments were performed prior to the initial administration of semaglutide (0.25 mg). After patients received semaglutide 1.0 mg for 180 days, their weights were measured. OHA, oral hypoglycemic agent.

Patients with T2DM who visited the Diabetes Care Center at Jinnouchi Hospital between June 2020 and October 2023, and who initiated treatment with a weekly dose of 1.0 mg semaglutide in addition to their existing medications, were retrospectively registered. Baseline assessments were performed before the initial weekly administration of 0.25 mg semaglutide. After the patients had received 1.0 mg semaglutide weekly for 180 days, their weights were measured on day 180 and the change in levels from baseline was recorded (Figure 1). Semaglutide 1.0 mg had the highest weight-reduction effect among the available GLP-1 RAs in Japan ^(7)^, and we therefore proactively introduced semaglutide 1.0 mg for all patients who were deemed to require further dose reduction and who provided consent. Written informed consent was obtained from all patients. This study was conducted in accordance with the Declaration of Helsinki, and the study protocol was approved by the Human Ethics Review Committee of Jinnouchi Hospital, Kumamoto, Japan (2023-11-3). This study was registered in the UMIN protocol registration system (identification: UMIN000054704).

### Blood sampling and measurement of clinical parameters

Measurement of glycated hemoglobin (HbA1c) and plasma glucose is routinely performed every month in patients with diabetes mellitus. Moreover, biochemical examinations of blood serum chemistry are usually performed before and after newly started medications, including GLP-1 RA, to determine drug-induced side effects. Blood samples for HbA1c, total cholesterol, low-density lipoprotein cholesterol, high-density lipoprotein (HDL) cholesterol, triglycerides, creatinine, estimated glomerular filtration rate, uric acid, aspartate aminotransferase, alanine aminotransferase, and γ-glutamyl transpeptidase were collected from the antecubital vein before GLP-1 RA therapy and analyzed in the Jinnouchi Hospital laboratory.

### Statistical analysis

The Shapiro-Wilk test was used to assess the normal distribution of continuous data. Data were expressed as mean ± standard deviation, and data with skewed distributions are expressed as the median value with interquartile range. Categorical data are presented as frequencies and percentages. Differences between paired samples were analyzed by the *t* test or Wilcoxon signed-rank test; the differences between two groups were analyzed by the *t* test, Mann-Whitney U test, or Fisher’s exact test as appropriate. It has been reported that a 7% weight loss improves blood pressure, lipids, and blood sugar levels ^(8)^. We therefore defined a greater response in weight reduction following 1.0 mg semaglutide per week as a >7.0% reduction in weight at 180 days from initiation of treatment. We analyzed this by simple logistic regression analysis, with greater weight reduction as the dependent variable and various parameters as independent variables. We investigated the factors clinically associated with greater body fat reduction following weekly 1.0 mg semaglutide using multivariate logistic regression analysis including significant factors in univariate logistic regression analysis. The Hosmer-Lemeshow test was conducted to investigate the goodness of fit of the logistic regression model. A receiver-operating characteristic (ROC) curve analysis was conducted to calculate a cutoff value of pretreatment levels of HDL-cholesterol for greater body-weight reduction. Statistical significance was set at p value < 0.05. Statistical analyses were performed by using SPSS version 23 (SPSS Inc., Chicago, IL, USA).

## Results

### Subjects

Of 250 patients with T2DM who were adequately managed with the administration of weekly semaglutide (0.25 mg in 61 patients; 0.5 mg in 93 patients; 1.0 mg in 96 patients), 96 patients managed with 1.0 mg semaglutide weekly were enrolled in this study. Table 1 shows the baseline characteristics of the participants, aged 56.7 ± 9.5 years, 58 of whom (60.4%) were male. Height was 164.9 ± 8.9 cm, weight was 82.7 (75.9-97.6) kg, body mass index was 30.8 (28.7-34.5) kg/m^2^, and HbA1c was 7.0 (6.3-7.8). Seventy patients (72.2%) switched from other GLP-1 RAs. Details of the switched GLP-1 RAs included three cases of lixisenatide 20 μg/day, two cases of liraglutide 0.6 mg/day, one case of liraglutide 0.9 mg/day, 12 cases of liraglutide 1.8 mg/day, and 52 cases of dulaglutide 0.75 mg/week.

**Table 1.** Clinical Characteristics of the Study Participants.

	Overall (N=96）	greater response group （N=23）	none-greater response group （N=73）	P value
Age (year)	56.7±9.5	59.2±10.7	55.9±9.0	0.1512
Male (%)	58 (60.4%)	13 (56.5%)	45 (61.6%)	0.8073
Height (cm)	164.9±8.9	163.8±7.7	165.2±9.2	0.5064
Weight (kg)	82.7 (75.9-97.6)	81.2 (71.9-89.6)	83.5 (77.6-98.1)	0.3846
BMI (kg/m^2^)	30.8 (28.7-34.5)	30.1 (28.1-31.9)	31.7 (28.9-34.7)	0.4783
Hemoglobin A1c (%)	7.0 (6.3-7.8)	6.6 (6.0-7.2)	7.1 (6.4-8.3)	0.0666
Aspartate aminotransferase (IU/L)	25 (19-35)	25 (20-36)	26 (19-35)	0.6619
Alanine aminotransferase (IU/L)	28 (20-42)	27 (17-37)	28 (23-43)	0.1955
γ-glutamyl transpeptidase (IU/L)	28 (20-49)	26 (23-51)	30 (20-48)	0.3606
Total cholesterol (mg/dL)	150 (132-182)	144 (132-188)	150 (131-181)	0.3936
HDL-cholesterol (mg/dL)	45 (38-51)	47 (43-58)	44 (37-51)	0.0058
LDL-cholesterol (mg/dL)	75 (59-91)	72 (58-97)	75 (59-87)	0.4271
Triglycerides (mg/dL)	141 (98-216)	130 (98-176)	141 (98-220)	0.2486
eGFR (ml/min/1.73 m^2^)	73.0 (61.0-83.3)	76.2 (53.2-83.8)	72.4 (61.4-83.3)	0.8153
Antidiabetic medicines (%)				
Insulin (combined)	37 (47.4%)	8 (34.8%)	29 (39.7%)	0.8072
Sulfonylureas (combined)	24 (24.7%)	4 (17.4%)	20 (27.3%)	0.4161
Glinides (combined)	4 (4.1%)	2 (8.7%)	2 (2.7%)	0.2417
DPP-4 inhibitor (switched)	7 (7.2%)	2 (8.7%)	5 (6.8%)	0.6715
GLP-1 receptor agonists (switched)	70 (72.2%)	12 (52.2%)	58 (79.5%)	0.0154
SGLT-2 inhibitor (combined)	81 (83.5%)	20 (87.0%)	61 (83.6%)	1.0000
Metformin (combined)	56 (57.7%)	15 (65.2%)	41 (56.2%)	0.4779
Thiazolidinedione (combined)	8 (8.2%)	1 (4.3%)	7 (9.6%)	0.6752
Alpha-glucosidase inhibitor (combined)	5 (5.2%)	3 (13.0%)	2 (2.7%)	0.0873

Data are represented as the mean ± SD, median [25th to 75th percentile range] BMI, body mass index; HDL, high-density lipoprotein; LDL, low-density lipoprotein; eGFR, estimated glomerular filtration rate; DPP-4, dipeptidyl peptidase-4; GLP-1 RA, glucagon-like peptide-1 receptor agonists; SGLT2, sodium glucose co-transporter 2

### Change in body weight after weekly treatment with 1.0 mg semaglutide

Weekly treatment with 1.0 mg semaglutide for 180 days significantly reduced body weight (−3.1 ± 4.8 kg, −2.7 [−5.5 to −0.6], p < 0.001) and HbA1c (−0.39% ± 1.23%, −0.1 [−0.8 to 0.2], p = 0.003).

### Comparison of clinical characteristics in patients with and without greater response in weight reduction

We defined the greater response group as having a weight reduction of more than 7.0% in relation to weekly treatment with 1.0 mg semaglutide. The number of patients in this greater response group was 23 (24.0%). The results of baseline clinical characteristics of the greater response group and non-greater-response group are shown in Table 1. The HDL-cholesterol level was significantly higher in the greater response group than in the non-greater-response group. Patients who switched from other GLP-1 RAs were significantly less frequent in the greater response group than in the non-greater-response group.

### Logistic regression analysis for presence of greater response in weight reduction after weekly treatment with semaglutide

Logistic regression analyses were performed to determine the presence of a greater response in weight reduction after weekly 1.0-mg semaglutide treatment (Table 2). A simple logistic regression analysis found pretreatment HDL-cholesterol levels (odds ratio [OR] 1.05; 95% confidence interval [CI] 1.01-1.10; p = 0.01) and presence of switch from other GLP-1 RAs (OR 0.28; 95% CI 0.10-0.76; p = 0.01) to be significantly related factors. Multivariable logistic regression analysis revealed that the pretreatment HDL-cholesterol level (OR 1.05; 95% CI 1.01-1.09; p = 0.02) was independently and significantly associated with greater weight reduction after weekly 1.0 mg semaglutide treatment, while a switch from other GLP-1 RAs (OR 0.31; 95% CI 0.11-0.87; p = 0.03) was independently and significantly associated with lesser weight reduction after weekly 1.0 mg semaglutide treatment.

**Table 2.** Logistic Regression Analysis for Presence of Greater Response in Weight Reduction after Weekly Treatment with Semaglutide.

	Simple regression			Multivariate regression		
	OR	95%CI for Odds ratio	P-value	OR	95%CI for Odds ratio	P-value
Age (per 1.0year)	1.0381	0.9862 - 1.0928	0.1527			
Sex (male)	1.2363	0.4782 - 3.1961	0.6616	1.1255	0.4016 - 3.1544	0.8221
Height (per 1.0 cm)	0.9818	0.9304 - 1.0360	0.5022			
Weight (per 1.0 kg)	0.9870	0.9584 - 1.0164	0.3816			
BMI (per 1.0 kg/m^2^)	0.9613	0.8629 - 1.0710	0.4744			
Hemoglobin A1c (per 1.0 %)	0.7091	0.4861 - 1.0344	0.0744			
Aspartate aminotransferase (per 1.0 IU/L)	0.9935	0.9651 - 1.0227	0.6589			
Alanine aminotransferase (per 1.0 IU/L)	0.9811	0.9530 - 1.0101	0.1994			
γ-glutamyl transpeptidase (per 1.0 IU/L)	1.0064	0.9926 - 1.0203	0.3656			
Total cholesterol (per 1.0 mg/dL)	1.0054	0.9931 - 1.0178	0.3907			
HDL-cholesterol (per 1.0 mg/dL)	1.0522	1.0103 - 1.0959	0.0140	1.0472	1.0059 - 1.0902	0.0248
LDL-cholesterol (per 1.0 mg/dL)	1.0067	0.9904 - 1.0232	0.4240			
Triglycerides (per 1.0 mg/dL)	0.9963	0.9902 - 1.0023	0.2270			
eGFR (per 1.0 ml/min/1.73 m^2^)	0.9977	0.9790 - 1.0168	0.8130			
Insulin (versus no use)	0.8092	0.3044 - 2.1513	0.6713			
Sulfonylureas (versus no use)	0.5579	0.1689 - 1.8423	0.3383			
Glinides (versus no use)	3.3810	0.4487 - 25.4747	0.2371			
DPP-4 inhibitor (versus no use)	1.2952	0.2340 - 7.1704	0.7670			
GLP-1 RA (versus no use)	0.2821	0.1042 - 0.7637	0.0128	0.3105	0.1106 - 0.8718	0.0264
SGLT-2 inhibitor (versus no use)	1.3115	0.3359 - 5.1211	0.6964			
Metformin (versus no use)	1.4634	0.5522 - 3.8786	0.4439			
Thiazolidinedione (versus no use)	0.4286	0.0499 - 3.6797	0.4399			
Alpha-glucosidase inhibitor (versus no use)	5.3250	0.8317 - 34.0942	0.0775			

Hosmer-Lemeshow goodness-of-fit χ2 for multivariate regression model was 4.58 with a P value of 0.810 OR, odds ratio; CI, confidence interval; BMI, body mass index; HDL, high-density lipoprotein; LDL, low-density lipoprotein; eGFR, estimated glomerular filtration rate; DPP-4, dipeptidyl peptidase-4; GLP-1 RA, glucagon-like peptide-1 receptor agonists; SGLT2, sodium glucose co-transporter 2

### ROC curve analysis of pretreatment levels of HDL-cholesterol for presence of greater response in weight reduction after weekly treatment with semaglutide

**Figure 2.**
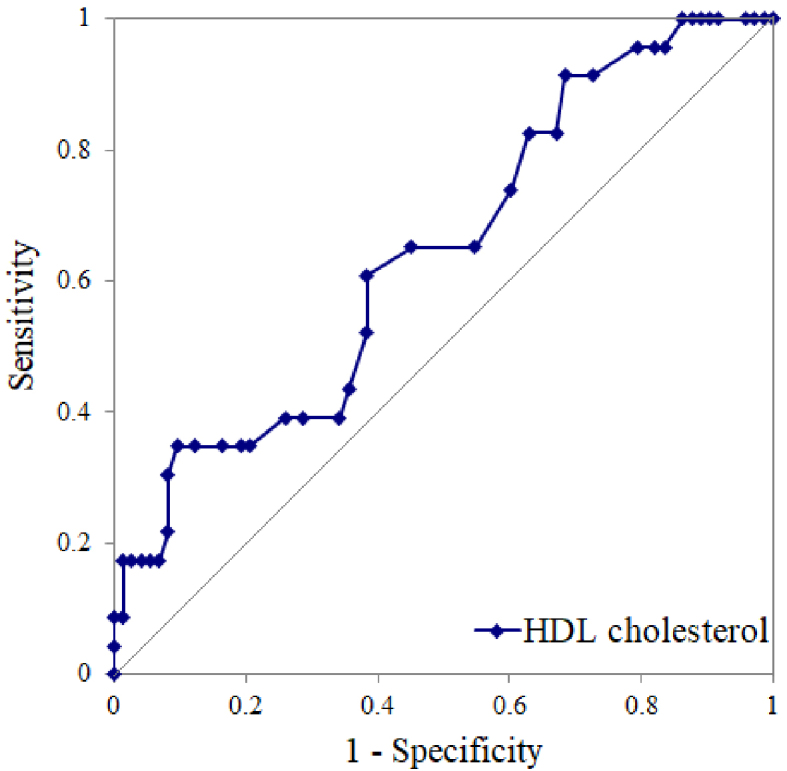
Receiver-operating characteristic curve analysis. Receiver-operating characteristic curve analysis was used to identify the optimal pretreatment level of HDL cholesterol for the presence of greater response in weight reduction after weekly treatment with 1.0 mg semaglutide. This cutoff value, based on the sensitivity and specificity values, was calculated as 46 mg/dL (area under the curve, 0.64; standard error, 0.05; 95% confidence interval, 0.51-0.77; *p* = 0.03). HDL, high-density lipoprotein.

We conducted a ROC curve analysis to calculate a cutoff value of pretreatment HDL-cholesterol levels for the presence of greater response in weight reduction after weekly treatment with 1.0 mg semaglutide, whereby we determined a cutoff of 46 mg/dL (area under the curve = 0.64; sensitivity, 61%; specificity, 62%; p = 0.03) (Figure 2). Among the greater responders (those with >7% body-weight reduction), a high OR of 2.5 was observed in patients with high HDL-cholesterol levels compared with those with low HDL-cholesterol levels (14 greater responders among 42 patients in the high HDL-cholesterol subgroup vs. 9 greater responders among 54 patients in the low HDL-cholesterol subgroup).

### Sub-analysis of weight-reduction percentage according to history of GLP-1 RA treatment

We investigated the percentage weight change after switching from all-GLP-1 RAs, liraglutide, and non-liraglutide GLP-1 RAs to weekly semaglutide 1.0 mg. Weight was significantly decreased in the all-GLP-1 RAs (−3.0 ± 5.1%, p < 0.01) and the non-liraglutide groups (−3.9 ± 4.8%, p < 0.01); however, there was no significant change in the liraglutide group (−0.1 ± 4.9%, p = 0.98). Significant weight reduction was observed in the group without prior GLP-1 RA treatment (−5.2 ± 5.5%, p < 0.01).

## Discussion

In the present study, pretreatment levels of HDL-cholesterol and switching from other GLP-1 RAs were significantly associated with weekly semaglutide-induced greater reduction in weight after 180 days of treatment with 1.0 mg semaglutide per week. We determined that the optimal HDL-cholesterol cutoff value for predicting weekly semaglutide-induced greater reduction in weight was 46 mg/dL.

Semaglutide may reduce body weight more effectively than the other GLP-1 RAs; only the group that switched from liraglutide to semaglutide showed no significant weight reduction. Semaglutide and liraglutide have similar peptide structures ^(9)^, and both have long half-lives and are considered more effective at suppressing appetite than other GLP-1 RAs ^(10)^. Regarding GLP-1 RAs other than liraglutide, switching to weekly semaglutide improved hyperglycemia and overweight ^(11), (12)^. Furthermore, semaglutide administration resulted in the greatest weight reduction, even compared with other glucose-lowering medications ^(13)^. Switching to weekly semaglutide may be beneficial when other GLP-1 RAs are unable to achieve sufficient weight reduction, given that further management of obesity is recommended to achieve better cardiovascular and renal outcomes ^(14)^.

HDL-cholesterol levels were significantly associated with weight reduction after GLP1-RA administration. Several factors and mechanisms are involved in the regulation of HDL-cholesterol levels and obesity, high leptin concentrations ^(15)^, and low adiponectin levels are closely related to low HDL-cholesterol levels and quality ^(16)^. Notably, leptin-deficient mice showed low serum HDL-cholesterol levels as a result of the downregulation of hepatic scavenger receptor class B member 1 ^(17)^. Regarding the relationship between leptin and GLP-1 RA, high plasma leptin concentrations weaken the effect of GLP-1 RA on food intake ^(18)^, and leptin-receptor-deficient obese and diabetic mice did not reduce their food intake after semaglutide administration. Leptin resistance, along with a high-fat diet, inhibits the effects of GLP-1 RA administration on food intake reduction ^(19)^. Conversely, the GLP-1 RA semaglutide enhances leptin sensitivity, although expression of the long-form leptin receptor is increased in obese animals ^(20)^. Notably, no previous studies have shown that HDL-cholesterol levels affect the weight-reduction effect of GLP-1 RAs, although the mechanism underlying this effect remains unclear. Measuring serum leptin concentrations and pretreatment HDL-cholesterol levels in clinical practice may help to predict the weight-reducing effects of semaglutide administration.

This study has several limitations. First, its retrospective design limited the ability of the study to establish causality and control for confounding factors. In addition, the study subjects were restricted to those who had adhered to semaglutide for 180 days. Second, no control group was included. Third, the sample size was relatively small, which may have limited the statistical power to detect any significant differences. Fourth, we were unable to consider any weight loss caused by GLP-1 RA used prior to switching. As a result, the mechanism underlying this relationship remains unclear and further prospective studies with larger sample sizes are needed. Fifth, this study did not measure blood leptin concentrations, although previous research has suggested that an increased blood leptin concentration may underlie GLP-1 resistance to body-weight reduction.

In conclusion, pretreatment HDL-cholesterol levels provide important information relating to weight-reduction expectations in patients with T2DM treated with weekly semaglutide 1.0 mg. Patients with T2DM who have never been administered GLP-1 RAs and have an HDL-cholesterol level of 46 mg/dL or higher may be the most suitable candidates for weight reduction with 1.0 mg semaglutide weekly.

## Article Information

### Conflicts of Interest

HJ received honoraria from Novo Nordisk, Sanofi, Eli Lilly, Mitsubishi Tanabe Pharma, and Otsuka Pharmaceutical. SS has received honoraria from AstraZeneca Pharmaceuticals and Ono Pharmaceutical. The authors declare that no other potential conflicts of interest exist in relation to this study.

### Acknowledgement

We thank Hugh McGonigle, from Edanz (https://www.jp.edanz.com/ac), for editing a draft of the manuscript.

### Author Contributions

Noboru Kurinami and Masafumi Takada contributed equally to the present study.

### Approval by Institutional Review Board (IRB)

The study protocol was approved by the Human Ethics Review Committee of Jinnouchi Hospital on November 6, 2023 (approval number: 2023-11-3).
